# Cognitive flexibility and entrepreneurial creativity: the chain mediating effect of entrepreneurial alertness and entrepreneurial self-efficacy

**DOI:** 10.3389/fpsyg.2023.1292797

**Published:** 2023-11-30

**Authors:** Xiaoling Yu, Xiuli Zhao, Yongxiong Hou

**Affiliations:** ^1^School of Marxism, South China Normal University, Guangzhou, China; ^2^Academy of Art and Design, Guangdong AIB Polytechnic, Guangzhou, China; ^3^School of Entrepreneurship, South China Normal University, Guangzhou, China

**Keywords:** entrepreneurial creativity, cognitive flexibility, entrepreneurial alertness, entrepreneurial self-efficacy, entrepreneurship education

## Abstract

Entrepreneurial creativity is an important part of entrepreneurs’ competency structure, and studies have been conducted to explore its impact on outcome variables like entrepreneurial performance, etc., but there are fewer studies on its antecedent variables at the individual level. In the relational model of entrepreneurial creativity, cognitive flexibility, entrepreneurial alertness, and entrepreneurial self-efficacy are included to construct a mediated use spss26.0 model, and data are collected from a research sample of 325 entrepreneurs for empirical analysis. The results indicate that: cognitive flexibility has a positive effect on entrepreneurial creativity; entrepreneurial alertness plays an independent mediating role between cognitive flexibility and entrepreneurial creativity, which is similar to how entrepreneurial self-efficacy plays the role between cognitive flexibility and creativity; entrepreneurial alertness and entrepreneurial self-efficacy then play a chain mediating role between cognitive flexibility and creativity. These findings reveal that three variables jointly influence entrepreneurial creativity, providing new theoretical and practical insights for understanding and enhancing entrepreneurial creativity. In addition, the study provides valuable guidance for entrepreneurship education and training, which can help entrepreneurs to better utilize their creativity and thus promote innovation and success in entrepreneurial activities.

## 1 Introduction

China’s economy is shifting from high-speed growth to a high-quality development stage, where the traditional development model of cost leadership and economies of scale is gradually declining, and the innovation-driven creativity economic model is becoming more and more important (Istudor, 2017). In the 21st century, Creative ideas and entrepreneurial activities have become indispensable elements of the economy (Belevskikh et al., 2019). In recent years, with the emergence and application of new technologies such as artificial intelligence and machine learning, robotic process automation (RPA), the Internet of Things (IoT), quantum computing, blockchain, etc., the time for technological turnover and iteration has shortened, while human creativity and innovation have become increasingly irreplaceable. The creativity economy is based on human creativity, and the realization of value transformation through the role of creativity to develop the economy and grow wealth is the essential performance of the knowledge economy (Chen, 2014). Entrepreneurs, as the main body of corporate innovation and intellectual resources, the structural composition and cultivation and enhancement of their creativity has become an important issue.

Creation means different things to different people and can be defined at different levels and disciplines (Bujor and Avasilcai, 2016). Guilford (1967) Belief that creativity is inherent in human beings and is not unique to so-called “geniuses,” that everyone in the world possesses varying degrees of creativity, and that divergent thinking is the kernel of the study of creativity. Amabile focused on product development in his research on creativity and based on this research proposed a three-component model of creativity arguing that creativity is comprised of skills, cognitive styles, and motivation within a given domain (Amabile and Pillemer, 2012). Stenberg, on the other hand, divided creativity into three dimensions such as intelligence, intellectual approach, and personality traits, and proposed the creativity system model. According to the creativity system model, he argues that the formation of creativity is similar to the development of natural selection in biological evolution, i.e., creativity arises in the process of interaction between the individual and the social and cultural environment in which the individual lives (Sternberg, 2006).

Du et al.’s (2021) research claims that creativity is essential for businesses to be successful, driving innovation, productivity, and growth, while also promoting a positive company culture and attracting talent. Shalley et al. (2004) research emphasizes the need for companies to intentionally foster creativity through policy, leadership, and the work environment. The interplay of factors affecting creativity also suggests the need for a holistic approach to capitalizing on corporate creativity (Fetrati et al., 2022). Entrepreneurial creativity enables the creativity embodied by the individual entrepreneur, and for entrepreneurial activity, entrepreneurial creativity is the most important personality trait for corporate innovation and is often the defining characteristic of entrepreneurs (Ahlin et al., 2014). Creativity plays an important role in the entrepreneurial process and enables entrepreneurs to compete in a dynamic environment. Researchers have extensively explored the important role of entrepreneurial creativity and have found that entrepreneurial creativity has a significant impact on the identification of entrepreneurial opportunities, the development of innovative products, and the formulation of marketing strategies (Ko and Butler, 2007).

For entrepreneurs, cognitive flexibility, entrepreneurial alertness and entrepreneurial self-efficacy are important sources of their entrepreneurial creativity (Hansen et al., 2011; Chang and Chen, 2020; Nisula and Olander, 2023). Cognitive flexibility reflects “awareness of the options and alternatives available in any given situation, a willingness to adapt flexibly to the situation and the confidence to respond flexibly” (Martin and Rubin, 1995), Cognitive flexibility affects the likelihood that an individual will engage in entrepreneurship, positively influencing entrepreneurial intentions (Dheer and Lenartowicz, 2019), High individual cognitive flexibility promotes startups’ strategic adaptability and helps them adapt to the external environment (Grégoire et al., 2011). In psychological research, cognitive flexibility is an important feature that helps humans pursue complex tasks such as multitasking and finding novel, adaptive solutions to changing needs (Ionescu, 2012). This psychological trait enables entrepreneurs to think about problems from different perspectives, to propose multiple solutions to problems, to avoid core rigidity, and to enhance the adaptability of entrepreneurship to be more sensitive to the fact that there are options and choices available in any given situation, and to be willing to flexibly adapt to the situation (Wang et al., 2021), thus entrepreneurs with high cognitive flexibility have higher creativity. Kirzner (2009) regards that entrepreneurial alertness to changing patterns of demand, prices, technological advances and other market changes is key to entrepreneurship, enabling entrepreneurs to be keenly attuned to market changes and opportunities that stimulate their creativity. Especially under conditions of increased use of social and business networking capabilities, the potential of entrepreneurial alertness as a driver of new business success is magnified (Adomako et al., 2018). Entrepreneurial self-efficacy, on the other hand, refers to the perceived ability to accomplish a specific task, and it is “not concerned with what a person has, but rather with what a person believes he can do with whatever resources he can focus on” (Bandura, 1999). Self-efficacy and entrepreneurial orientation have a direct positive effect on firm performance, while creativity and firm performance are fully mediated by entrepreneurial orientation (Khedhaouria et al., 2015).

However, although cognitive flexibility, entrepreneurial alertness, and entrepreneurial self-efficacy have been recognized as important sources of entrepreneurial creativity, the relationship between them and how they work together to influence the process of entrepreneurial creativity has not yet been adequately investigated theoretically and empirically. This study is based on Albert Bandura’s Social Cognitive Theory and Icek Ajzen’s Theory of Planned Behavior and will focus on answering the following questions: First, a mediation model on the key antecedents of entrepreneurial creativity was constructed by clarifying the links between cognitive flexibility, entrepreneurial alertness, entrepreneurial self-efficacy and entrepreneurial creativity. We responded to a number of scholarly initiatives (Mumin et al., 2013), which is, moving beyond a discussion of the role of creativity and focusing on more than just the performance impact that creativity brings to a business. Second, how is entrepreneurial creativity enhanced? This study reveals the positive influence relationship between cognitive flexibility and entrepreneurial creativity through empirical research, and constructs a chain mediation model to explore the indirect influence of cognitive flexibility on entrepreneurial creativity through entrepreneurial alertness and entrepreneurial self-efficacy. These findings will inspire entrepreneurs to remain competitive in their entrepreneurial practices, as well as provide guidelines for entrepreneurship education. To address this research need, this paper proposes nine hypotheses and conducts an empirical study through a survey of 325 entrepreneurs. Our findings confirm the validity of these hypotheses, reveal how cognitive flexibility, entrepreneurial alertness, and entrepreneurial self-efficacy work together to influence entrepreneurial creativity, and provide new theoretical and practical insights for understanding and enhancing entrepreneurial creativity, and for developing 21st-century-oriented competencies such as creativity, innovative thinking, and entrepreneurial spirit.

## 2 Literature review and research hypotheses

### 2.1 Entrepreneurial creativity

Creativity leads socio-cultural, economic and technological progress and is a key factor in the advancement of political and spiritual civilization (Paulo et al., 2009). Sternberg (2017) defines creativity as the production of a novel, surprising, and compelling (high quality) idea or product, and for a person or product to be truly creative, it must have all three of these characteristics at the same time. The process of creativity involves the use of one or more production functions to create novel products or outputs. Agents involved in the process are also important, as their unique capabilities and access to resources (e.g., materials and technology) contribute to the development of innovative and creative ideas (Burns et al., 2015). Entrepreneurial creativity therefore involves the ability of entrepreneurs to combine previously separated elements in new ways to create improved or entirely new products, services, processes or practices. By doing so, they add value to what was previously available in the market or production environment (Mumin et al., 2013). This echoes Schumpeter’s doctrine of entrepreneurship as “creative destruction” (Schumpeter, 1912). Entrepreneurial Creativity Positively Moderates Entrepreneurial Behavior and Psychological Well-Being of Startup Entrepreneurs (Wang et al., 2021). A large body of academic literature has demonstrated the importance of creativity for the start-up and survival of businesses in complex and competitive environments (Heunks, 1998; Hansen et al., 2011). In these studies, the role of individual entrepreneurial or employee creativity in value creation has been widely discussed, but the role of entrepreneurial creativity and its antecedents has received less systematic theoretical attention (Mumin et al., 2013). A number of previous studies have focused on the effects of personal and situational characteristics on creativity, and a theoretical overview of this issue has been presented (Shalley et al., 2004). There are also a few studies that have attempted to explore the specific factors that influence creativity, for example, one study noted a significant correlation between the positive emotions of founding entrepreneurs and their creativity, which in turn is significantly and positively correlated with firm-level innovation (Baron and Tang, 2011). It has also been found that individual traits such as openness, curiosity, and risk-taking of entrepreneurs are positively correlated with creativity (Tony et al., 2014). Emotions and moods also have a significant impact on entrepreneurial creativity (Susan et al., 2012), for example, positive moods and emotions contribute to entrepreneurial creativity. Creativity self-efficacy is another important influence, i.e., an individual’s perception and assessment of his or her own creativity ability, and creativity self-efficacy is closely related to entrepreneurs’ creativity performance (Khedhaouria et al., 2015).

In summary, entrepreneurial creativity as a key driver of entrepreneurial success has attracted widespread attention in academia and practice, but most studies focus on the outcome variables of entrepreneurial creativity, and even though there are some studies that explore the key personality traits that determine creativity, the empirical results are inconclusive in terms of the existing literature (Mumin et al., 2013), and the attention to entrepreneurial creativity antecedents still has a lot of room for growth. Considering that entrepreneurial creativity is an important factor for start-ups to discover resources, improve performance, and promote business growth and sustainability, it is crucial to explore what factors influence entrepreneurial creativity.

### 2.2 Cognitive flexibility

Cognitive flexibility refers to how a person reconfigures his or her mental resources by integrating external evidence into the reasoning process (Cañas et al., 2003). A report by the World Economic Forum (2016) identified cognitive flexibility as one of the ten most important skills predicted for entrepreneurial success. Cognitive flexibility has three important components; first, it implicitly involves a learning process; second, it involves the adaptation of cognitive processing strategies; and finally, this adaptation will occur as a response to new and unexpected changes (Romero-Ferreiro et al., 2022). Individuals with high cognitive flexibility are able to overcome concepts and ideas that are functionally rigid due to typecasting in a variety of complex ways, creating relationships between them that are not easily detectable (Dajani and Uddin, 2015), thus to find viable solutions to seemingly contradictory problems and combine and reorganize knowledge gathered from different sources in new ways (Martin and Rubin, 1995), greater variety of knowledge affects creativity and innovation and the ability to implement new ideas, thereby facilitating rapid innovation and change (Shane, 2000).

Cognitive flexibility reflects an entrepreneur’s ability to switch between different modes of thinking (Martin and Rubin, 1995), and individuals with high cognitive flexibility are able to flexibly switch from one stimulus, processing, or mental mode to another when necessary (Vartanian, 2009). In exploring cognitive flexibility in entrepreneurship research, researchers have identified cognitive flexibility as a core heuristic in entrepreneurial thinking that helps find new solutions to strategic challenges in new ventures (Michael Haynie et al., 2012). Cognitive flexibility enables entrepreneurs to switch between different cognitive processing styles, which facilitates decision-making, especially in environments with high levels of complexity and uncertainty. Cognitive flexibility promotes strategic adaptation in startups, helping them to adapt to the external environment and respond to the dynamic competitive strategies of their competitors, while at the same time taking the lead in environmental change, creating the right environment for the business to thrive, influencing entrepreneurs to creatively excel in stressful environments, and thus improving the resilience and performance of the business (Grégoire et al., 2011).

Entrepreneurs with high cognitive flexibility have high levels of creativity. Cognitive flexibility emphasizes the entrepreneur’s ability to think about problems from different perspectives, to come up with multiple solutions to problems, to avoid core rigidity and to enhance entrepreneurial adaptability. Entrepreneurs with cognitive flexibility realize that there are options and choices available in any given situation and are willing to flexibly adapt to the situation (Wang et al., 2021). From an opportunity perspective, cognitive flexibility can motivate entrepreneurs to proactively scan and search for information as well as check for profitable opportunities. Individuals with high cognitive flexibility have higher cognitive demand, are less anxious about new or controversial situations, and enjoy exploring new information and ideas (Martin et al., 2011). Based on the above analysis, we propose Hypothesis 1.

**H1: Cognitive flexibility has a positive effect on entrepreneurial creativity**

### 2.3 Entrepreneurial alertness

Entrepreneurial alertness is the degree to which decision makers anticipate entrepreneurial opportunities in the current and future business environment, and is a key factor influencing the identification, construction and implementation of entrepreneurial opportunities (Kirzner, 2009). In recent years, the concept of entrepreneurial alertness has become a key concept in entrepreneurship research (Obschonka et al., 2017), and researchers have conducted rich explorations around entrepreneurial alertness and firm performance, factors influencing entrepreneurial alertness, entrepreneurial alertness and entrepreneurial opportunity identification, entrepreneurial alertness and entrepreneurial behavior. In the existing research, two main types of entrepreneurial alertness with different connotations are presented, an entrepreneurial alertness focusing on a market-clearing mechanism focused on economic equilibrium, and an entrepreneurial alertness focusing on the cognitive and psychological nature of the participants in the entrepreneurial process (Lanivich et al., 2022). An exploration of the key antecedents of entrepreneurial alertness based on an information processing perspective suggests that entrepreneurial personality, training, and experience can all influence entrepreneurial alertness (Tang et al., 2012). Sharma’s (2019) study also points out again that perceiving and searching for information, cognitive ability, personality factors, knowledge and experience all influence entrepreneurial alertness, with cognitive ability playing a central role in the construct of alertness. Social cognitive theory is one of the more established cognitive theories that has been used to explain entrepreneurial alertness (Chavoushi et al., 2021). Social cognitive theory places special emphasis on the influence of individual cognition and motivation on individual behavior. With the continuous development of Internet technology and the increasing transparency of information, entrepreneurs need to scrutinize and monitor various market elements and constantly look for market opportunities (Vaghely and Julien, 2010). A high level of cognitive flexibility enables individuals to overcome cognitive inertia, which will deepen the entrepreneur’s awareness of the relationship between existing cognitive schemas and new information, help the entrepreneur integrate valid information and filter invalid information, and enable the entrepreneur to flexibly reconfigure existing cognitive schemas (Eisenhardt et al., 2010). Understanding and reconstructing cognitive schemas helps entrepreneurs stay alert to market changes (Tang, 2008), enabling entrepreneurs to activate schemas quickly and accurately in an ambiguous scenario and notice the emergence of opportunities. Based on the above analysis, we propose Hypothesis 2.

**H2: Cognitive flexibility has a positive effect on entrepreneurial alertness**

Entrepreneurial alertness directly affects strategic change decisions and organizational performance (Roundy et al., 2018), and increased levels of entrepreneurial alertness are associated with improved start-up performance (Adomako et al., 2018). Entrepreneurial alertness is a distinguishing characteristic that distinguishes entrepreneurs from non-entrepreneurs (Kirzner, 1979). Entrepreneurs with high entrepreneurial alertness have the ability to identify objectively available business opportunities in the marketplace more readily than others. Entrepreneurial alertness directly predicts opportunity recognition, while prior knowledge indirectly affects opportunity recognition through its effect on entrepreneurial alertness (Li et al., 2015). The effect of entrepreneurial alertness on entrepreneurial intentions is positively moderated by competitive traits, while the effect of entrepreneurial intentions on entrepreneurial behavior is positively moderated by proactive personality. This implies that for people with competitive and proactive personalities, their entrepreneurial alertness is more likely to be transformed into entrepreneurial intentions, and their entrepreneurial intentions are more likely to be transformed into actual entrepreneurial behavior (Neneh, 2019). This study argues that entrepreneurial alertness reflects entrepreneurs’ ability to perceive information about opportunities and resources in the environment and to conduct value-creating activities based on it, which is a dynamic and evolving set of perceptual abilities. Entrepreneurs with high entrepreneurial alertness have higher creativity, show sharper market insights than others, and are able to identify new market needs and deploy resources in a timely manner to satisfy them. This study expands the scope of research on entrepreneurial alertness to the relationship with entrepreneurial creativity, which complements and enriches the discussion on entrepreneurial alertness in the field of entrepreneurship research.

Based on the above, hypotheses 3 and 4 are proposed:

**H3: Entrepreneurial alertness has a positive effect on entrepreneurial creativity**

**H4: Entrepreneurial alertness mediates the relationship between cognitive flexibility and entrepreneurial creativity**

### 2.4 Entrepreneurial self-efficacy

Bandura (1977) defines “self-efficacy” as an individual’s ability and skill to accomplish specific tasks and perform a particular job. This definition describes how actions, behaviors, perceptions, cognitions, and environments interact in a self-motivated manner. Individuals with high levels of self-efficacy for a particular job or task are more likely to persevere with that task than individuals with low self-efficacy (Bandura, 1999). With the growing emphasis on entrepreneurial thinking and action, research on entrepreneurial self-efficacy has become a key psychological concept in entrepreneurship research and has been found to influence entrepreneurial motivation, intentions, behaviors, and performance, as well as being an important target outcome for entrepreneurship training and education (Newman et al., 2019). The core idea of entrepreneurial self-efficacy reflects an individual’s assessment of his or her own abilities and judgment of the level of confidence in accomplishing entrepreneurial tasks.

As entrepreneurs’ beliefs about their ability to perform different entrepreneurial roles and tasks (Chen et al., 1998). As analyzed above, cognitive flexibility influences the individual’s way of thinking and behavioral choices, and higher cognitive flexibility makes entrepreneurs strive to level up and maintain a high level of attention when solving problems. When individuals have good cognitive flexibility, it is easy for them to perceive the generation of new information and accurately judge entrepreneurial opportunities, thus enabling entrepreneurs to maintain confidence in their own judgment and ability, and to enhance their sense of entrepreneurial self-efficacy. On this basis, this study proposes hypothesis 5.

**H5: Cognitive flexibility has a positive effect on entrepreneurial self-efficacy**

A number of studies have combined social cognitive theory in order to reveal the impact of self-efficacy on entrepreneurial intention and entrepreneurial behavior (Pihie and Bagheri, 2013; Bagheri et al., 2022), and entrepreneurial self-efficacy has a positive effect on entrepreneurial decision making and moderates entrepreneurial risk propensity and entrepreneurial decision making (Ma and Yan, 2015). Sense of efficacy increases the likelihood of becoming a fledgling entrepreneur and creating a running business (Cassar and Friedman, 2009). Entrepreneurial self-efficacy is a dynamic concept that changes over time under the influence of experience, in which self-ratings of specific entrepreneurial knowledge and skills are more stable than general levels of self-efficacy (Krecar and Coric, 2013). Overall, when entrepreneurs have high self-efficacy, they have more self-motivation, more courage and conviction to face uncertainty in entrepreneurial activities, and more creativity, in contrast to entrepreneurs with low entrepreneurial self-efficacy who may be more conservative and comfortable with the *status quo*, assess the entrepreneurial environment more negatively, and have a higher likelihood of withdrawing from entrepreneurial activities. Based on this, the following hypotheses are proposed:

**H6: Entrepreneurial self-efficacy has a positive effect on entrepreneurial creativity**

**H7: Entrepreneurial self-efficacy mediates the relationship between cognitive flexibility and entrepreneurial creativity**

Specific characteristics of entrepreneurs who exhibit positive actions in entrepreneurial activities include positive goals and visions, entrepreneurial orientation, positive task strategies and action plans, positive social network strategies, positive feedback on failed behaviors, and active learning (Frese and Gielnik, 2023). Positive action is the key to influencing the creation and growth of new ventures, and entrepreneurs with high entrepreneurial alertness tend to act positively and promote entrepreneurial self-efficacy by forming clear goals and visions, action plans, building social networks, and learning from failures and sticking to their goals. Based on this, we propose the last two hypotheses, Hypothesis 8 and Hypothesis 9:

**H8: Entrepreneurial alertness has a positive effect on entrepreneurial self-efficacy**

**H9: Entrepreneurial alertness and entrepreneurial self-efficacy as chain mediators between cognitive flexibility and entrepreneurial creativity**

**FIGURE 1:**
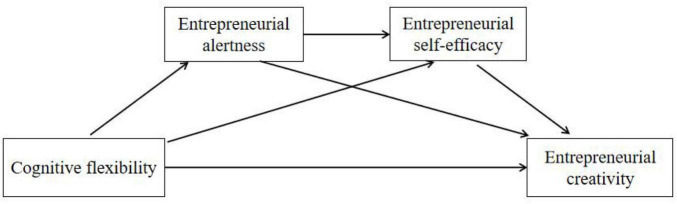
Research model map.

In summary, this study proposes a model as shown in Figure 1. Cognitive flexibility has a direct positive effect on entrepreneurial creativity, while entrepreneurial creativity can be enhanced through entrepreneurial alertness and entrepreneurial self-efficacy as independent mediators respectively, in addition to the chain mediation of entrepreneurial alertness and entrepreneurial self-efficacy.

## 3 Materials and methods

### 3.1 Participants

This study utilized both paper and electronic questionnaires to conduct the survey and data collection. The target respondents of the survey are mainly entrepreneurs in Guangdong Province, including entrepreneur trainees of Guangdong Province’s training course for improving the competence of start-up business operators, entrepreneurs selected for the “New Economy, She Leads” Guangzhou Innovation and Entrepreneurship Pioneer, entrepreneurs of new businesses in innovation and entrepreneurship competitions such as “Zongchuang Cup,” “Internet Plus” and “Winning in Guangzhou,” and entrepreneurs of new businesses through entrepreneur associations and other channels. “Internet+,” “Winning in Guangzhou” and other innovation and entrepreneurship competitions, as well as entrepreneurs connected to entrepreneurs through entrepreneurs’ associations and other channels, and the data collection period is from August 2022 to November 2022. The questionnaires were distributed in a variety of ways: entrepreneurs in Guangdong Province’s training courses for improving the competence of startup operators filled out the questionnaires by arranging time to do so during the course of the lectures and training; entrepreneurs in the “New Economy, She Leads” Guangzhou Innovation and Entrepreneurship Pioneers were distributed the questionnaires at the same time as in-depth interviews were conducted; entrepreneurs in the “Zongchuang Cup,” “Internet+,” “Winning in Guangzhou” and other innovation and entrepreneurship competitions, the research team contacted the entrepreneurs to distribute and collect the questionnaires through the organizing committee; and entrepreneurs connected through entrepreneur associations and other channels, with the help of the connectors to fill out the questionnaires. There are also entrepreneurs who are connected through entrepreneurs’ associations and other channels, and the research team will directly contact the entrepreneurs to distribute and collect the questionnaires after the connectors help to communicate with them initially and provide their contact information.

A total of 510 questionnaires were distributed in this study, 423 were returned, and 98 invalid questionnaires were excluded, including interruptions, short completion time, vacancies, or invalid information. Finally, 325 valid questionnaires were obtained, with an effective recovery rate of 63.73%. From the demographic characteristics of the sample, the gender distribution is 47.7% (*n* = 154) male and 52.6% (*n* = 171) female; the age distribution is 42.8% (*n* = 139) below 25 years old, 42.8% between 26 and 30 (*n* = 139) years old, 5.5% (*n* = 18) between 31 and 35 years old, 2.8% (*n* = 9) between 36 and 40 years old, and 6.1% (*n* = 20) above 40 years old; the education distribution is 32.9% (*n* = 107) junior college and below, 56.6% (*n* = 184) bachelor’s degree, 8.9% (*n* = 29) master’s degree, and 1.5% (*n* = 5) doctor’s degree; the company size is 10.2% (*n* = 33) with less than 5 people, 38.2% (*n* = 124) with 6–20 people, 46.8% (*n* = 152) with 21–50 people and 4.9% (*n* = 16) with more than 51people; and the number of years of enterprise founding accounted for 1 year or less, accounting for 37.2% (*n* = 121), 1–3 years accounted for 48.9% (*n* = 159), and more than 3 years accounted for 13.8% (*n* = 45).

### 3.2 Measurements

The variables measured in this study were all based on relevant domestic and international mature scales, and the linguistic descriptions of the scales were moderately adjusted according to the research context and purpose. All scales were based on the Likert five-point scale method, where 1 represents complete non-compliance, while 5 represents complete compliance, respectively.

#### 3.2.1 Cognitive flexibility

Cognitive flexibility was measured using a scale developed by Martin and Rubin (1995), which consists of 12 items, including 4 reverse items. They pointed out that cognitive flexibility consists of three aspects: conscious thinking about options and alternatives in any situation, flexibility and willingness to adapt to the situation, and entrepreneurial self-efficacy to use different methods to cope with various problems. Scales include “I can express an idea in many different ways,” “I try to avoid new and unusual situations (reversed),” “I feel like I never make real decisions (reversed),” “I can find workable solutions to seemingly unsolvable problems,” and so on. The Cronbach’s alpha coefficient for this scale was tested to be 0.753.

#### 3.2.2 Entrepreneurial alertness

Entrepreneurial alertness was measured using a scale developed by Boso. The scale was derived from the Entrepreneurial Alertness Scale developed by Tang et al. (2012). They categorized entrepreneurial alertness into three dimensions, namely, search and scanning, association and connection, and assessment and judgment, from an information processing perspective. Subsequently, Boso et al. (2019) revised the scale to obtain the Entrepreneurial Alertness Scale with good reliability and validity. The scale consists of 11 items, including “I regularly read news, magazines, or trade publications for new information,” “I can find connections between seemingly unrelated information,” “I can often find connections between information systems that were previously unrelated,” “I can often find connections between information systems that were previously unrelated,”and so on. The Cronbach’s alpha coefficient for this scale was tested to be 0.923.

#### 3.2.3 Entrepreneurial self-efficacy

Entrepreneurial self-efficacy was measured, based on the Ozgen (2003) scale, using a scale containing five items: “I believe that it is easier for me to start a business” “I believe that I can choose an industry with potential to start a business” “I believe that my knowledge, abilities and qualities are conducive to my success in starting a business” “I believe that my life and work experiences are conducive to my success in starting a business” “If I start a business, I have a good chance of succeeding.” The Cronbach’s alpha coefficient value for this scale is 0.855.

#### 3.2.4 Entrepreneurial creativity

Entrepreneurial creativity was measured using the scale developed by Ahlin et al. (2014). The scale has good currency and has been used by scholars in China (Xiu’e and Kun, 2018), showing good reliability and validity, so this scale was used in this study as a tool to measure entrepreneurial creativity. The scale includes “I am a creative person,” “I spend time every week or every day thinking of new ideas,” “I have a lot of novel ideas,” “I often look for new solutions even when they are not necessary” “My ideas are often new and unique” “New solutions often pop into my head even when they are not necessary” “I can easily come up with proposals to improve the situation I can easily come up with suggestions to improve the current situation” “I often find problems that others cannot” 8 items. The Cronbach’s alpha coefficient value for this scale is 0.915.

#### 3.2.5 Control variables

Control variables. In order to avoid the possible interference of other variables on the explanatory variables of the model, the entrepreneurs’ age, gender, education, company size (number of employees), and time of establishment were controlled.

### 3.3 Reliability and validity testing

#### 3.3.1 Reliability testing

SPSS 26 software tool was used to test the reliability of cognitive flexibility, entrepreneurial alertness, entrepreneurial self-efficacy, and entrepreneurial creativity of the samples, and the alpha values of all the variables resided between 0.753 and 0.923, which were greater than the threshold value of 0.7, indicating that the variables of the present study were reasonably and reliably measured, and had sufficient consistency and stability to be used in subsequent empirical studies.

#### 3.3.2 Common method bias testing

The data of the study were obtained from the subjects’ self-reports, which may be subject to common method bias. In order to avoid common method bias, the study was controlled accordingly in the research procedures, such as setting polygraph questions and using anonymous responses. Meanwhile, in order to make the study more rigorous, the commonly used Harma’s single-factor test to test for common method bias before data analysis. The results showed that the first factor with a characteristic root rule greater than 1 explained 38.06% of the overall variance, which is below the 40% criterion (Zhou and Long, 2004). There are no serious common method biases.

## 4 Results

### 4.1 Preliminary analyses

The means, standard deviations, correlation coefficients, and internal consistency coefficients of the variables involved in this study are shown in Table 1, which shows that (1) cognitive flexibility (CF) is significantly and positively correlated with entrepreneurial alertness (EA) (*r* = 0.497, *p* < 0.01), entrepreneurial self-efficacy (ESE) (*r* = 0.471, *p* < 0.01), and entrepreneurial creativity (EC) (*r* = 0.480, *p* < 0.01); (2) entrepreneurial alertness is significantly and positively correlated with entrepreneurial self-efficacy (*r* = 0.735, *p* < 0.01) and entrepreneurial creativity (*r* = 0.714, *p* < 0.01) were significantly positively correlated; (3) entrepreneurial self-efficacy was significantly positively correlated with entrepreneurial creativity (*r* = 0.671, *p* < 0.01). The above results are consistent with the proposed hypotheses and offer the possibility of further testing the hypotheses subsequently.

**TABLE 1:** Means, standard deviations, and correlation coefficients for variables (*N* = 325).

	Means	S.D.	Gender	Age	Education	Years	People	CF	EA	ESE	EC
Gender	0.53	0.5	1								
Age	1.89	1.126	0.118*	1							
Education	1.79	0.661	0.129*	0.382**	1						
Years	1.77	0.676	-0.055	-0.396**	-0.089	1					
People	2.47	0.768	0	0.095	0.263**	0.107	1				
CF	3.7338	0.41047	-0.088	0.194**	0.091	-0.204**	0.009	**0**.**753**			
EA	3.7712	0.55051	-0.012	0.223**	0.036	-0.241**	-0.035	0.497**	**0**.**923**		
ESE	3.744	0.54631	0.077	0.186**	0.077	-0.206**	0.012	0.471**	0.735**	**0**.**855**	
EC	3.7338	0.60503	-0.004	0.109*	0	-0.137*	0.012	0.480**	0.714**	0.671**	**0**.**915**

*, **Represent respectively *p* < 0.05, *p* < 0.01. The bold numbers on the diagonal in the table are the internal consistency coefficients for the scale. CF, cognitive flexibility; EA, entrepreneurial alertness; ESE, entrepreneurial self-efficacy; EC, entrepreneurial creativity.

### 4.2 Relational modeling and chained mediation effect tests

Due to the high correlation between the core explanatory variables in this article, in order to avoid bias caused by multicollinearity, this article conducted multicollinearity tests on the data before regression. The test results indicate that the variance inflation factors (VIFs) for cognitive flexibility, entrepreneurial alertness, and self-efficacy are 1.372, 2.319, and 2.245, respectively, indicating that the VIF values of all explanatory variables are far below their acceptable upper limit of 10.0, indicating that there is no multicollinearity problem among the explanatory variables in this study.

Due to the high correlation between the core explanatory variables in this article, in order to avoid bias caused by multicollinearity, this article conducted multicollinearity tests on the data before regression. The test results indicate that the variance inflation factors (VIFs) for cognitive flexibility, entrepreneurial alertness, and self-efficacy are 1.372, 2.319, and 2.245, respectively, indicating that the VIF values of all explanatory variables are far below their acceptable upper limit of 10.0, indicating that there is no multicollinearity problem among the explanatory variables in this study.

**FIGURE 2:**
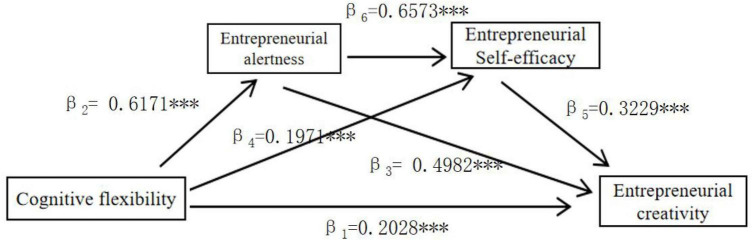
Relation model map. ***Significant correlation at the 0.001 level (two-tailed).

Based on the findings of this study, Figure 2 depicts the interplay between cognitive flexibility, entrepreneurial alertness, entrepreneurial self-efficacy, and entrepreneurial creativity.

#### 4.2.1 Direct effect testing

The path coefficient of Cognitive Flexibility → Entrepreneurial Creativity (R^2^ = 0.5761, *F* = 53.6819, *p* < 0.001) is β_1_ = 0.2028,*t* = 3.1531, *p* < 0.001, which indicates that Cognitive Flexibility significantly and positively affects Entrepreneurial Creativity, i.e., there is a direct effect of Cognitive Flexibility and Entrepreneurial Creativity, and Hypothesis 1 that Cognitive Flexibility has a positive effect on Entrepreneurial Creativity holds.

#### 4.2.2 Mediation effect test

To explore the role of entrepreneurial vigilance, entrepreneurial self-efficacy in the relationship between cognitive flexibility and entrepreneurial creativity. This study conducted chained mediation effect tests through SPSS macro program PROCESS edited by Hayes (2013). A Bootstrap sample size of 5000 was chosen to test the role of entrepreneurial alertness, entrepreneurial self-efficacy in the relationship between cognitive flexibility and entrepreneurial creativity at 95% confidence interval (CI) with gender, age, education, years of experience in the company, and size of the company as control variables, respectively, and the results of the Bootstrap test are shown in Table 2.

**TABLE 2:** Mediation effect test results.

	Effect path	Boot effect	SE	95% CI	
				**LLCI**	**ULCI**
Direct effect	Cognitive Flexibility → Entrepreneurial Creativity	0.2028	0.0643	0.0763	0.3294
Mediatingeffect	Cognitive Flexibility → Entrepreneurial Alertness → Entrepreneurial Creativity	0.3074	0.0626	0.1913	0.4374
	Cognitive Flexibility → Entrepreneurial Self-efficacy → Entrepreneurial Creativity	0.0637	0.0271	0.0188	0.1253
	Cognitive Flexibility → Entrepreneurial Alertness → Entrepreneurial Self-efficacy → Entrepreneurial Creativity	0.1310	0.0358	0.0637	0.2005
	aggregate intermediary effect	0.5021	0.0621	0.3818	0.6241
Total effect	Direct effect + Mediating effect	0.7049

(1) The path coefficient of Cognitive Flexibility → Entrepreneurial Alertness (*R*^2^ = 0.2778, *F* = 20.3845, *p* < 0.001) is the path coefficient β_2_ = 0.6171, *t* = 9.3071, *p* < 0.001, which indicates that Cognitive Flexibility significantly positively affects Entrepreneurial Alertness, and Hypothesis 2 that Cognitive Flexibility has a positive impact on Entrepreneurial Alertness is valid; the path coefficient of Entrepreneurial Alertness → Entrepreneurial Creativity (*R*^2^ = 0.5761, *F* = 53.6819, *p* < 0.001) has a path coefficient of path coefficient β_3_ = 0.4982, *t* = 7.9963, *p* < 0.001, indicating that entrepreneurial alertness significantly and positively affects entrepreneurial creativity, and Hypothesis 3 that entrepreneurial alertness has a positive effect on entrepreneurial creativity holds true; and the value of mediating effect between cognitive flexibility → entrepreneurial alertness → entrepreneurial creativity is (ind1) 0.3074, SE = 0.0626, 95% confidence interval of Bootstrap = 5000 is [0.1913, 0.4374], excluding 0, indicating that the mediating effect of entrepreneurial alertness between cognitive flexibility and entrepreneurial creativity is significant, and Hypothesis 4 Entrepreneurial Alertness plays a mediating role between Cognitive Flexibility and Entrepreneurial Creativity is established.
(2) The path coefficient of cognitive flexibility → entrepreneurial self-efficacy (*R*^2^ = 0.5661, *F* = 59.0752, *p* < 0.001) is β_4_ = 0.1971, *t* = 3.4207, *p* < 0.001, indicating that cognitive flexibility significantly and positively affects entrepreneurial self-efficacy, and Hypothesis 5 Cognitive Flexibility has a positive impact on entrepreneurial self-efficacy is established; the path coefficient of entrepreneurial self-efficacy → entrepreneurial creativity (*R*^2^ = 0.5761, *F* = 53.6819, *p* < 0.001) has a path coefficient of β_5_ = 0.3229, *t* = 5.2447, *p* < 0.001, indicating that entrepreneurial self-efficacy significantly and positively affects entrepreneurial creativity, and hypothesis 6 that entrepreneurial self-efficacy has a positive effect on entrepreneurial creativity holds true; and the value of the mediating effect between cognitive flexibility → entrepreneurial self-efficacy → entrepreneurial creativity (ind2) is 0.0637, with a Bootstrap = 5000 has a 95% confidence interval of [0.0188, 0.1253] excluding 0, indicating that entrepreneurial self-efficacy has a significant mediating effect between cognitive flexibility and entrepreneurial creativity, and hypothesis 7 entrepreneurial self-efficacy has a mediating effect between cognitive flexibility and entrepreneurial creativity is established.
(3) The path coefficient of Entrepreneurial Alertness → Entrepreneurial self-Efficacy (*R*^2^ = 0.5661, *F* = 59.0752, *p* < 0.001) was β_6_ = 0.6573, *t* = 15.2133, *p* < 0.001, which indicated that Entrepreneurial Alertness was able to significantly and positively affect entrepreneurial self-efficacy, and that Hypothesis 8, Entrepreneurial alertness has a positive impact on entrepreneurial self-efficacy, was valid; the chained mediation effect value (ind3) of entrepreneurial alertness and entrepreneurial self-efficacy in the cognitive flexibility and entrepreneurial creativity with a chain mediation effect value (ind3) of 0.1310 and a 95% confidence interval of [0.0637, 0.2005] excluding 0 for Bootstrap = 5000, indicating that Entrepreneurial alertness and entrepreneurial self-efficacy have a significant chain mediation effect between cognitive flexibility and entrepreneurial creativity, Hypothesis 9 entrepreneurial alertness and entrepreneurial self-Efficacy play a chain mediating role between cognitive flexibility and entrepreneurial creativity as a chain mediating effect between cognitive flexibility and entrepreneurial creativity was established.

## 5 Discussion and limitations

### 5.1 Discussion

Based on the social cognitive theory and theory of planned behavior, this study constructs a theoretical framework and chain mediation model from cognition to competence “Cognitive Flexibility - Entrepreneurial Alertness - Entrepreneurial Self-Efficacy - Entrepreneurial Creativity” theoretical framework and chain mediation model, and explored how cognitive flexibility indirectly affects entrepreneurial creativity through entrepreneurial alertness and entrepreneurial self-efficacy, as well as the direct effect of cognitive flexibility on entrepreneurial creativity. After empirical testing of 325 data samples, nine hypotheses were verified and the following conclusions were drawn:

(1) Cognitive flexibility has a direct positive effect on entrepreneurial creativity

As predicted in Hypothesis 1, entrepreneurs’ cognitive flexibility positively affects entrepreneurial creativity, and entrepreneurs with higher cognitive flexibility scores have higher entrepreneurial creativity. This phenomenon can be explained in two ways. On the one hand, individuals with high cognitive flexibility have more novel and useful ideas, are curious about new things, like to explore new information and ideas, and can innovate and change quickly, and new information and ideas can be transformed and innovated quickly in individuals with high cognitive flexibility, so this type of individuals also have higher entrepreneurial creativity. On the other hand, the specificity of cognitive flexibility contributes to entrepreneurial creativity. In the process of entrepreneurship, entrepreneurs will encounter problems such as entrepreneurial financing, social support, entrepreneurial guidance, etc., which require good social skills and problem-solving abilities, and entrepreneurs with high cognitive flexibility are good at solving problems in these social fields. Therefore, entrepreneurs with higher cognitive flexibility have greater entrepreneurial creativity. This finding confirms the importance of cognitive flexibility in creativity and entrepreneurship.

From an individual’s perspective, increasing entrepreneurial creativity can solve many specific problems in the entrepreneurial process and daily social activities of entrepreneurs and help them to succeed in their entrepreneurial career. From the school’s point of view, combining elements of social creativity training with entrepreneurship education activities and curricula will help to improve entrepreneurs’ entrepreneurial performance. Although this study is an initial exploration of the relationship between cognitive flexibility and entrepreneurial creativity, its findings on their relationship provide empirical support for personal entrepreneurship development and entrepreneurship education in schools. Future research could also develop specific pedagogical content for social creativity development based on the issues involved in the field of entrepreneurship and further test the effectiveness of social creativity in the field of entrepreneurship.

(2) Mediating effects of entrepreneurial alertness, entrepreneurial self-efficacy

In addition to the overall chain of intermediaries, each individual intermediary link in the model is worth discussing. Entrepreneurs with high entrepreneurial alertness have the ability to identify objectively available business opportunities in the marketplace more readily than others. Entrepreneurial alertness directly predicts opportunity recognition, while prior knowledge indirectly affects opportunity recognition through its effect on entrepreneurial alertness (Li et al., 2015). Entrepreneurial alertness reflects the entrepreneur’s ability to perceive information about opportunities and resources in the environment and conduct value creation activities based on it, which is a dynamic and constantly evolving set of perceptual abilities. Entrepreneurs with high entrepreneurial alertness have higher creativity, show sharper market insight than others, and are able to discover new market demand in time and deploy resources to meet the market demand in a timely manner. Entrepreneurial alertness is a key path to enhance entrepreneurial creativity, and entrepreneurs’ entrepreneurial creativity can be enhanced by increasing their entrepreneurial alertness. Entrepreneurial alertness mediates the relationship between cognitive flexibility and entrepreneurial creativity.

The indirect effect of entrepreneurial passion on entrepreneurial intentions through entrepreneurial self-efficacy is significant and positive, and social support moderates the indirect effect of entrepreneurial passion on entrepreneurial intentions through entrepreneurial self-efficacy (Neneh, 2019). When entrepreneurs have high entrepreneurial self-efficacy, they have more self-motivation and more courage and conviction to face uncertainty in entrepreneurial activities. Entrepreneurial self-efficacy mediates the relationship between cognitive flexibility and entrepreneurial creativity.

Positive action is key to the creation and growth of new ventures, and entrepreneurs with high entrepreneurial alertness tend to act positively and promote entrepreneurial self-efficacy by forming clear goals and visions, action plans, building social networks, and learning from failures and sticking to their goals. Entrepreneurs can increase cognitive flexibility and enhance entrepreneurial alertness to increase entrepreneurial self-efficacy, which in turn increases entrepreneurial creativity, and entrepreneurial alertness and entrepreneurial self-efficacy have a chain mediating effect between cognitive flexibility and entrepreneurial creativity.

The study explores the antecedents of entrepreneurial creativity, the centerpiece of Schumpeterian innovation, and fills a gap in research on the factors influencing entrepreneurial creativity. It expands the mechanism by which cognitive flexibility affects entrepreneurial creativity, including the direct effect and chain-mediated effect; and provides theoretical basis for the development of innovation and entrepreneurship education by improving cognitive flexibility, entrepreneurial alertness and entrepreneurial self-efficacy. It not only enriches the theoretical research on cognitive flexibility, entrepreneurial alertness, entrepreneurial self-efficacy and entrepreneurial creativity, but also provides new perspectives and methods for entrepreneurship education and entrepreneurial practice.

### 5.2 Implications

(1) Strengthening entrepreneurship education. In the age of knowledge and intelligence, creativity, innovation and entrepreneurship have become the main forces driving economic and social development. At present, universities and colleges in many countries have carried out various forms of entrepreneurship education, and existing studies have also shown that entrepreneurship education has a significant impact on entrepreneurial self-efficacy, entrepreneurial creativity, entrepreneurial ability, entrepreneurial willingness and entrepreneurial behavior. Based on the findings of this study, we suggest that entrepreneurship education should widely cover the basic methodology of innovative creativity and creativity development in addition to teaching the relevant knowledge and skills of entrepreneurship management. Entrepreneurship education should be committed to meeting the requirements for the quality cultivation of innovative talents, set up basic courses aimed at enhancing the skills related to intrinsic innovation motivation and creativity within individuals, such as the frontiers of innovation design, innovation management, etc., and in the process, fully take into account the individual’s professional field and traits Considering the important roles of cognitive flexibility, entrepreneurial alertness, and entrepreneurial self-efficacy in the field of entrepreneurship and its own malleability, the training of the relevant content should be incorporated into the entrepreneurship education curriculum, enhance entrepreneurial creativity through systematic curriculum teaching and social training, and help entrepreneurs clarify and enhance their entrepreneurial willingness and ability. At the same time, we suggest that entrepreneurship education should not only be carried out in colleges and universities, but should realize displacement and coverage from colleges and universities to enterprises. Enterprises can learn from colleges and universities to embed entrepreneurship education modules with their own characteristics in the training system, so as to encourage employees to carry out internal innovation and entrepreneurship, and to realize the interface between entrepreneurship education in colleges and universities and enterprises’ own practice. By changing and optimizing individual cognitive styles and ability levels, cognitive flexibility, entrepreneurial alertness and entrepreneurial self-efficacy can be enhanced, entrepreneurship can be improved, and entrepreneurial creativity can be realized.
(2) Optimizing entrepreneurship policies. This study is also a certain degree of validation of China’s existing dual-creation policy, which promotes the joint formation of a positive social atmosphere to encourage entrepreneurship by multiple parties. In the face of the booming development and emerging challenges of creative innovation and entrepreneurial activities, the government should adjust its policy formulation strategy, change the past practice of intensively issuing a large number of policy documents in a short period of time, pay more attention to the formulation of mature institutionalized policies, further refine the core content of creative innovation and entrepreneurship policies on the basis of the existing types of policies such as guidelines, action plans, planning outlines, management methods, etc., and issue the field’s regulations, administrative rules and other institutionalized policies. At the same time, the use of policy tools to promote the integration and development of innovation and entrepreneurship should be strengthened, and all-round support should be provided for all aspects and development stages of innovation and entrepreneurship. Society as a whole should develop a situation that supports entrepreneurship and encourages creativity, while having a higher tolerance for failure. Through better policy orientation, entrepreneurs should be given more support and encouragement to enhance their cognitive flexibility, entrepreneurial alertness and sense of entrepreneurial self-efficacy, and in the process, entrepreneurial creativity should be continuously enhanced to create a better environment for innovation and creativity.

### 5.3 Limitations

Despite the innovative findings of this study, some limitations remain. First, this study relied primarily on questionnaires, which may have been affected by self-report bias. Future research could consider using more diverse data collection methods, such as experiments, interviews, or case studies. Second, the sample of this study was mainly from one country, China, which may limit the generalizability of the findings. Future research could expand the sample to consider more diverse cultural and economic backgrounds. Finally, this study mainly focuses on the mediating mechanism of cognitive flexibility in influencing entrepreneurial creativity, without further exploring the effects of the corresponding moderating variables, and there may be other important variables that deserve attention. Future research could attempt to examine the contextual effects of other environmental factors in this process to deepen the findings of this study.

## Data availability statement

The original contributions presented in this study are included in this article/supplementary material, further inquiries can be directed to the corresponding author.

## Ethics statement

Ethical approval was not required for the studies involving humans because review and approval were not required for the study on human participants in accordance with the local legislation and institutional requirements. The studies were conducted in accordance with the local legislation and institutional requirements. The participants provided their written informed consent to participate in this study. Written informed consent was obtained from the individual(s) for the publication of any potentially identifiable images or data included in this article.

## Author contributions

XY: Conceptualization, Methodology, Resources, Validation, Writing – original draft, Writing – review and editing. XZ: Data curation, Formal analysis, Supervision, Writing – review and editing, Methodology, Software, Validation. YH: Conceptualization, Data curation, Formal analysis, Funding acquisition, Investigation, Project administration, Resources, Supervision, Visualization, Writing – original draft, Writing – review and editing.
